# Genetic Algorithm Application in Optimization of Wireless Sensor Networks

**DOI:** 10.1155/2014/286575

**Published:** 2014-02-16

**Authors:** Ali Norouzi, A. Halim Zaim

**Affiliations:** Computer Engineering Department, Istanbul University, 34320 Istanbul, Turkey

## Abstract

There are several applications known for wireless sensor networks (WSN), and such variety demands improvement of the currently available protocols and the specific parameters. Some notable parameters are lifetime of network and energy consumption for routing which play key role in every application. Genetic algorithm is one of the nonlinear optimization methods and relatively better option thanks to its efficiency for large scale applications and that the final formula can be modified by operators. The present survey tries to exert a comprehensive improvement in all operational stages of a WSN including node placement, network coverage, clustering, and data aggregation and achieve an ideal set of parameters of routing and application based WSN. Using genetic algorithm and based on the results of simulations in NS, a specific fitness function was achieved, optimized, and customized for all the operational stages of WSNs.

## 1. Introduction 

WSNs are constituted of small sensors with specialized applications and limitations designed for specific purposes. The applications are divided into military, commercial, and medical applications. Among military applications are communication, command, and intelligence defense networks. Health care system for disables in remote areas, smart environment for the elderly, physicians, and medical staff communication networks, and patient surveillance systems are some of medical applications. Moreover, there is a wide range of commercial applications including security systems, fire safety systems, environment pollution monitor systems (chemical, microbial, and nuclear pollutions), vehicle tracking, supervising and controlling systems, traffic control system, and natural disasters studies (e.g., earthquake and flood) [1]. Wide range of applications has resulted in development of variety of protocols which include plenty of flexible parameters. At any rate, some parameters, due to their wide range of utilization, can be found in several applications (as common parameters) and of great importance. Wireless sensor networks use mobile energy sources and rechargeable batteries, and due to technological limitations, these batteries can supply energy for a short period of time. Thus, optimum utilization of energy in such networks is of great importance [2].

**Figure 1:**
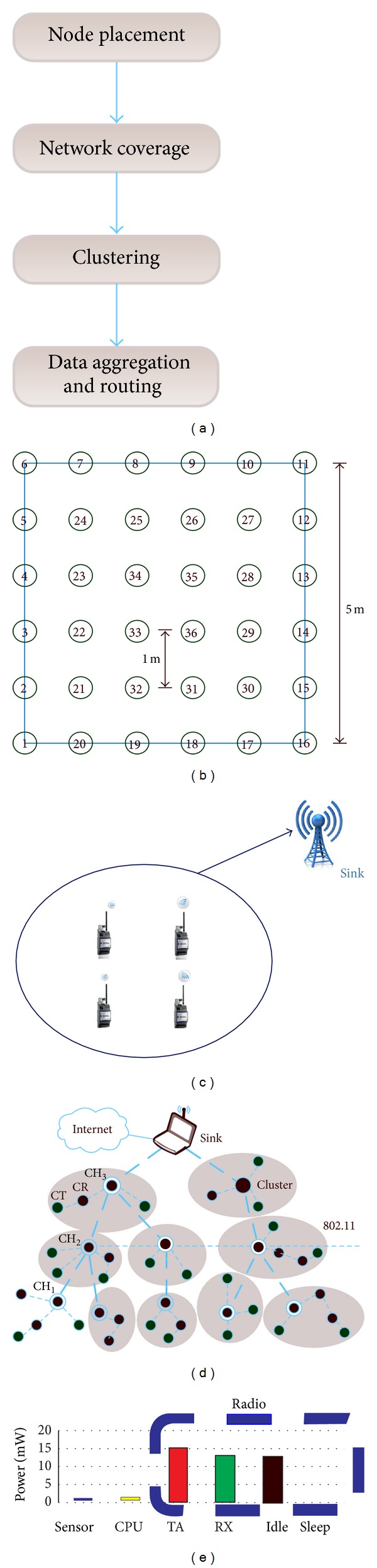
(a) Main operatioanl stages of WSNs. (b) Grid layout of WSN. (c) Network coverage stage. (d) Clustering in WSNs. (e) Energy consumption in different states of WSNs.

Necessity of data integrity in WNSs due to support continuous and permanent communication among the sensors has made the lifetime another important parameter in WSNs. The present study surveys some specific parameters throughout different operational stages of WSNs. In general, operational stages of classic WSNs are divided into node placement, network coverage, clustering, and data aggregation.  Figure 1(a) pictures general classification of the main operational stages of WSNs.

An important stage before establishment of a WSN is “node placement.” Generally, there are several types of node distributions in WSNs including regular, random, and grid distributions. Under grid layout, the distance between each node can be estimated. An example of grid layout is pictured in Figure 1(b) and, clearly, the gap between the nodes is fixed.

The decision about type of layout depends on the expected application, so that nodes for military purposes are usually scattered by airplanes over military zones, while in case of underwater sensors, regular distribution is adopted and grid layouts are usually used for urban networks (Figure 1(b)).

Poisson's distribution is useful for modeling different types of random phenomena; it generates an estimate of binomial probabilities. In addition to an estimator distribution, Poisson's distribution is a useful probability model for the events that happen randomly whether in time or place. The distribution is usually used for detailed study on and simulation of wireless networks.

The next stage is to connect the sensors based on the range of service. As mentioned before, among different features of sensors, radio range and service domain are key factors. Taking into account the required area coverage, the best layout must be adopted to reach the best quantity and quality of the services. Ineffective layout means waste of energy and financial resources.

Clustering is another main operation, which plays a key role in WSN optimization. By clustering, the sensor nodes are divided into groups known as division cluster. Each cluster has a cluster head that aggregates data from the nodes in the cluster and forwards the data to the sink directly or step by step using other clusters' heads. Therefore, the nodes may reduce their communication heading compared with the situation when data are forwarded directly to the sink. That is, clustering is an effective approach to attenuate load between sensor nodes.

Figure 1(d) illustrates nodes clustering in WSNs. Clustering is a way to save more energy and increase lifetime of the sensors in WSNs. The technique also has other advantages such as improved security, less extra data, and improved scalability of the network. To achieve better performance, different protocols can be used depending on the application.

Babamir and Norouzi proposed an efficient aggregate signcryption scheme to maximize the security of data in a kind of wireless medical network named the disconnected or unattended wireless sensor network [3]. Also in other work, they proposed another new secure scheme in which various security goals such as confidentiality, authentication and integrity. In addition, the aggregation process of their scheme reduces the space and communication overheads both for sensors and sink. The proposed technique efficiently enables the sensors and sinks to protect, verify, and recover all the related data [4].

The protocols are reliable ways to increase lifetime of the networks, although they cause more energy consumption by the cluster heads. Therefore, to increase lifetime of the network, cluster heads must be reelected during each period of cluster layout. In spite of the fact that the protocols ensure implementation of an effective clustering algorithm, they fail to guarantee adoption of the best node as cluster head.

Through optimization, the algorithms may attenuate energy consumption to a great extent and consequently improve efficiency and lifetime lifetime of the network.

Eventually, the transfer of data and queries between the main stations, information sinks, or events is another important issue in WSNs. A simple process for transfer of data is the direct transfer of data between the node and base station. The single-step oriented process is too costly; the more the distance between the node and base station, the more energy is needed and consequently the shorter the lifetime of the network.

Another process for transfer of data is multistep oriented transfer for a specific radius. This process saves considerable deal of energy and lessens collision in the network to a great deal, although, depending on the place of using routing mechanisms, they have some limitations.

Main reason that makes researchers more interested in the issue data gathering and routing stages is the considerable energy consumed at this stage.  Figure 1(e) pictures required energy in every states of the WSN. Clearly, the highest energy consumption is by radio communication. Therefore, more detailed studies on this stage hold great promises to optimize WSN concerning energy consumption and lifetime of the network [2, 5, 6].

Improvement of the parameters mentioned above eventuates in an optimized WSN. There are variety of methods to this end, such as fuzzy theory, neural networks, and evolutionary algorithm and thanks to its better results for larger scale networks and the fact that it generates final formula at the end, genetic algorithm is more common. The availability of final formula makes the algorithm more useful and helpful for human users. Thus, the present study uses genetic algorithm for optimization and customization of the networks [7].

This paper is organized as follows; Section 2 gives a brief description of genetic algorithm. Sections 3, 4, 5 and 6 present our proposed fitness function in node placement, network coverage, clustering, data aggregation, and details of algorithms, respectively. Finally, Section 7 presents conclusions and suggestions for future projects.

## 2. Genetic Algorithm 

**Figure 2:**
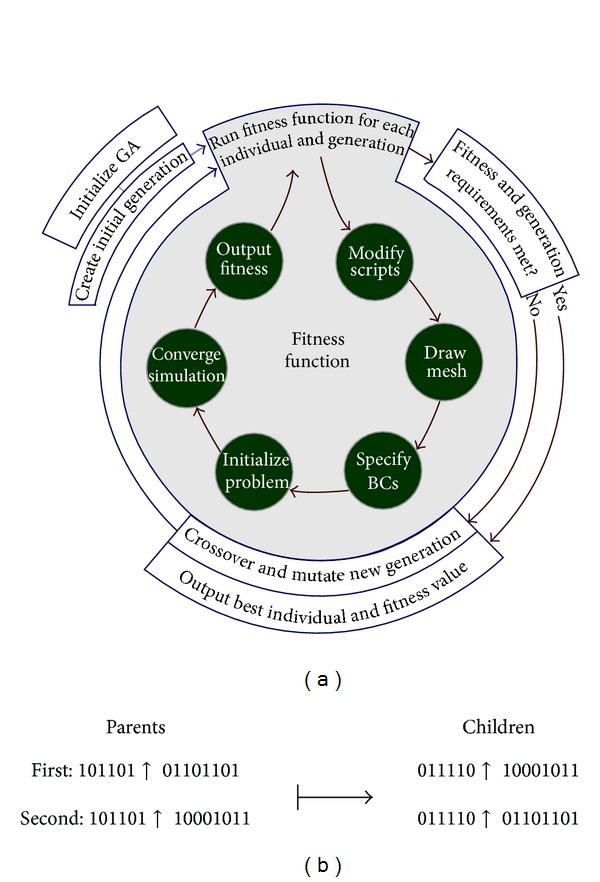
(a) The general scheme of GA mechanism. (b) Single point method at random point 6.

Also known as a global heuristic algorithm, a generic algorithm estimates an optimal solution through generating different individuals [8]. Focused fitness function is one of procedures of the algorithm. Following section describes the fundamental parts of a generic algorithm.  Figure 2(a) indicates the general scheme of genetic algorithm mechanism.

### 2.1. Initialization

The genetic algorithm starts with an elementary population comprised of random chromosomes which includes genes with a sequence of 0 s or 1 s. Afterward, the algorithm leads individuals to achieve an optimum solution by the way of repetitive processes including crossover and selection operators. There are two ways to develop a new population [9]: steady-state GA and generational GA. In the case of the former, one or two members in the population are replaced and at the same time, the generational GA replaces all the generated individuals of a generation.

### 2.2. Fitness

Under the genetic algorithm, the fitness function, by definition, is a process for scoring each chromosome based on their qualification. The assigned score is a trait for continuation of further reproduction. Dependence to problem by the fitness function is considerable, so that in case of some problems, it is not possible to define the problem. Naturally, individuals are permitted to go to the new generation based on their fitness score. Therefore, the score dictates the fate of individuals.

### 2.3. Selection

During every successive generation, a new generation is developed through adopting members of the current generation to mate on the bases of their fitness. The individuals with higher fitness score have higher chance for being selected, the process which results in preferential adoption of the best solution. Majority of the functions include a stochastically designed element for adopting small number of less fit individuals for sake of keeping diversity in the population [10]. Among the many selection methods, Roulette-Wheel is adopted to differentiate proper individuals with the probability of

(1) $$\begin{array}{c} P_{i}=\frac{F_{i}}{\sum_{j=1}^{n}F_{i}}, \end{array}$$

where *F*_*i*_ and “*n*” are the fitness chromosome and the size of population, respectively. According to the Roulette-Wheel, each individual is assigned a value between 0 and 1.

### 2.4. Crossover

The crossover or reproduction process constitutes the major step toward production. Indeed, sexual reproductive process by wich inherited characteristics are transferred from one generation to the next generation,is simulated. In the reproduction process, crossover process adopts a couple of individuals as the parents through breeding selection process. The process continues to reach the desired size in the new population. Generally, several crossover operations take place, each of which with different aims. The easiest way is single point, where a random point is adopted to divide the role of the patents. One example of mating by two chromosomes in single point way is pictured in Figure 2(a).

Figure 2(b) represents two children that are from a single set of parents. The bit sequence of the offspring duplicates one parent's bit sequence until the crossover point. Afterward, the bit sequence of the other parent is replicated as the second part of children.

### 2.5. Fitness Parameters in Wireless Sensor Network

The fitness of a chromosome determines the extent to which the consumption of energy is minimized and coverage is maximized. In what follows, some important fitness parameters in WSN are discussed.

(1) Direct distance to base station (DDBS): it refers to the sum of direct distance between all sensornodes and the BS represented by *d*_*i*_ as

(2) $$\begin{array}{c} \text{DDBS}=\sum_{i=1}^{m}d_{i}, \end{array}$$

where “*m*” stands for the number of nodes. Clearly, consumption of energy, reasonably, is subject to the number of nodes and for large WSN the energy is extreme. Moreover, DDBS is acceptable for smaller networks where number of close nodes is not considerable.

(2) Cluster based distance (CD): The total CHs and BS distances and the sumof the distance between the determined member nodes and their cluster heads (3).

(3) $$\begin{array}{c} \text{CD}=\left(\sum_{i=1}^{n}\left(\sum_{j=1}^{m}d_{ij}\right)+D_{is}\right), \end{array}$$

where “*n*” and “*m*” stand for the number of clusters and related members, respectively; “*d*_*ij*_” represents the distance between a node and its CH; and “*D*_*is*_” stand for distance between the CH and the BS. The solution suits networks with a large number of widely-spaced nodes. Higher cluster distance leads to higher energy consumption. For minimization of energy consumption, the CD must not be too large [11]. The density of the clusters is controlled by adopting this measurement, while density is the count of nodes in each cluster.

(3) Cluster-based distance-standard CDSD: instead of an average cluster distance, standard derivation measures the changes of distances of the cluster. CDSD is a function of the placement of sensor nodes (random or deterministic). There are clusters with different sizes in random placement so that a SD within a specified variation in the cluster distance is acceptable. If so, the differences in cluster distance is not zero, while the variation must be adopted based on the deployment of information [12]. At any rate, under deterministic placement with uniform distribution of node positions, cluster distance change must be minimized. Generally, changes of uniform cluster-based distances show that the network is poor, which is not the case when the nodes are placed randomly:

(4) $$\begin{array}{c} \mu =\frac{\sum_{i=1}^{n}d_{c}}{n},\\ \text{SD}=\sqrt{\sum_{i=1}^{n}{\left(\mu -d_{c}\right)}^{2}}, \end{array}$$

“*µ*” in equations (4) stands for the average of the cluster distances, which is the standard SD formula for obtaining cluster distance variation.

(4) Transfer energy (*E*): it stands for the amount of consumed energy required for transferring all the collected data to the BS. Let “*m*” be the number of associated nodes in a cluster; then, *E* is obtained by

(5) $$\begin{array}{c} E=\sum_{i=1}^{n}\left(\sum_{j=1}^{m}e_{jm}+m*E_{R}+e_{i}\right), \end{array}$$

where “*e*_*jm*_” stands for the required energy to transfer data from a node to the corresponding CH. Thus, the first term in the summation of “*i*” stands for the total consumption of energy for transfer of aggregated data to CHs. Moreover, the second term in the summation “*i*” pictures the total required energy to collect data from members, and finally, “*e*_*i*_” stands for the required energy for transmission from the cluster head to the BS.

(5) Number of transmissions (*T*): in general, the BS dictates the number of transmissions that occurs at every monitoring period. This measure is obtained based on the conditions and the energy level of the network; therefore, “*T*” stands for a long time stage for which the superior optimum solution for maximizing and an inferior solution for minimization are acceptable. The quality of the best solution or chromosome determines the performance of previous GA-based solutions.

In what follows, using genetic algorithm, a fitness function formula to improve each main operational aspects of WSNs (e.g., node placement, network coverage, clustering, and data aggregation) is introduced and discussed. In other words, fitness functions are mainly used to improve energy consumption and lifetime parameters. Simulation results confirmed improvement of the protocols.

## 3. Node Placement in Wireless Sensor Network

The placement of sensor nodes on a monitored field may influence the general performance of the network. Taking into account the placement of nodes in the field, there are three main categories of placement of nodes in a network including the deterministic node placement (grid), the semi- deterministic node placement (e.g., Biased Random), and the nondeterministic (stochastic) node placement (e.g., Simple Diffusion and Random). Long range transmission by sensor nodes is not energy efficient as it needs more energy than a linear function of transmission distance does. Clearly, node density is just one element in network topology as the placement of the node is another key factor. The placement of nodes influences the capacity of a network to correctly sense an event as well as the number of possible disjoint paths towards the sink(s).

Under the deterministic node placement, the nodes are placed on exact, preset points on a grid or in specific parts of the grid. Commonly, deterministic or controlled node placement dictates the type of nodes, the environment that nodes will be placed, and the application. Thus, in Sensor Indoor Surveillance Systems or Building Monitoring application nodes must be placed manually [13]. Under semi- deterministic placement, on the other hand, individual nodes are positioned in a nondeterministic way on the grid (e.g., random) which covers the areas nodes must be spread. That is, microscopic and macroscopic ways of placement of nodes are nondeterministic and deterministic, respectively.

**Figure 3:**
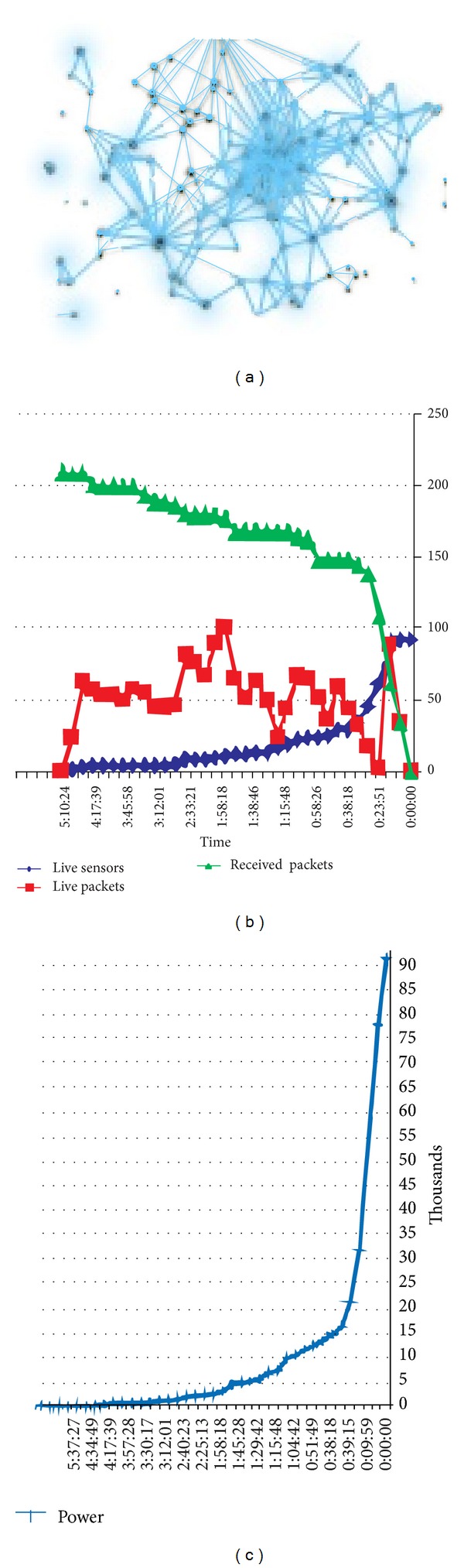
(a) Node placement scheme. (b) Comparison between number of available sensors, live and received packets existing in the network. (c) Comparison between mount of power and lifetime of network.

To make sure that network runs with the highest feasible performance, the nodes are positioned on the campus network. Along with balanced energy consumption of all nodes, a preferred node placement protocol is supposed to supply a better network throughput through attenuating contention of channel and collision of packet under high load. An instance of a node placement scheme is pictured in Figure 3(a).

The common advantages of proper sensor propagation in WSNs are listed below [14].

*Scalability.* A high number of nodes can be deployed in the network; this is suitable when transmissions between the nodes are not unlimited.

*Collision Reduction.* Since the cluster head (CH) functions as a coordinator, a limited number of nodes gain access to the channel and cluster members and head communicate locally.

*Energy Efficiency.* High energy consumption is a consequence of the periodic relocation. Still, duties of CH may be distributed among all other nodes through periodic relocation, which results in lower energy consumption.

*Low Cost.* The excess costs can be avoided by deploying sensors at proper locations.

*Routing Backbone.* The data collected by cluster members are aggregated in CH and sent to the sink. Thus, using a little route-thru traffic and routing backbone with enough efficiency one can build the network.

### 3.1. Problem Statement

Among the main aspects of improvement of performance for wireless sensor networks, node placement is one to name. Here, we discuss layout optimization of wireless sensor networks (WNNs). All the sensor nodes located in the environment should have a connection with high energy level nodes. For transmitting aggregated data, the nodes relay from environment to base or ground to a satellite. Sensor nodes are not efficient choice for long-term transmission as their energy consumption is a super linear function of the distance the data that is transmitted.

In this part, we assume that communication range of the sensor is fixed and the new Intelligent Node Placement Protocol in Wireless Sensor Networks using generic algorithm is introduced. The two competing objectives—total sensor coverage and lifetime of the network, are optimized in the proposed framework for WSNs. Thanks to the genetic algorithm, the proposed approach results in a solution where the sensing range is covered with a minimum number of nodes while optimum energy consumption is met.

### 3.2. The Proposed Fitness Function

Calculation of a minimum number of nodes is required in the algorithm. The next step is to evaluate connectivity of the network. This improves architecture of network. In addition, the algorithm takes the connection radius of applied nodes into account. This demonstrates that formula is flexible while different kinds of networks are measured. A fitness function based on the extension of area under coverage is introduced in what follows. It is mainly aimed to realize an optimum solution to cover wider area while efficiency of energy consumption is preserved. Afterward, connectivity of the nodes is examined by prime and Dijkstra algorithms. The coverage and the lifetime of the network are the two main objectives under consideration. The former is obtained by the area of the unit of the disk, which is obtained by radius *R* centered at each sensor. As per (6), the area of the union is normalized by the total area [15]

(6) coverage ={⋃i=1nRxi,yi2∴|(xi,yi)−(xi−1,yi−1)|=(0,0)⋃i=1nRxi,yi2−rxi,yi2∴o.w,

where →*r*_*x*_*i*_,*y*_*i*__ = |(*x*_*i*_, *y*_*i*_) − (*x*_*i*−1_, *y*_*i*−1_)|.

Quint et al. introduced the formula (7) for obtaining the energy required for converting a set of purpose points known as purpose function [16]

(7) $$\begin{array}{c} \min\_f=\sum_{i\in s}^{}\left(\varepsilon +d_{i}\right)*y_{i}+\sum_{i\in D}{\text{NC}}_{j}*h_{j}. \end{array}$$

In (7), the constant *ε* is the required energy to set up a node and *d*_*i*_ represents the cost of routing between upstream through downstream. This mount is computed before launching the program by using the Dijkstra algorithm. This value is a kind of penalty for remote nodes. NC is the surcharge of uncovering points; *y*_*i*_ and *h*_*j*_ show the activation status of node *i* and covering status of point *j*, respectively. Consider

(8) $$\begin{array}{c} \text{fitness}-\text{function}=\frac{\min\_f_{i,j}}{\text{coverag}{\text{e}}_{i,j}}. \end{array}$$

The proposed fitness function to take into account both the proposed coverage formula and energy procedure is represented in (8). Ration of energy level and amount of energy required to achieve the proper network are obtained by the formula.

### 3.3. Evaluation and Simulation Results

The experiments were conducted with 200 nodes (*N*), a network of 100 ∗ 100 m^2^ in extent (*M*), and a BS at 200 m from the network. The simulation parameters are listed in Table 1. As the communication medium between the sensors is binary, optimization of WSN connectivity space is significantly nonlinear. Considerable effects on the two objectives (network disconnection might be the case) is induced by trivial movement of the sensors. Thanks to high efficiency for nonlinear objectives, GA was used in optimization [15].

The GA parameters in the environment simulation are listed in Table 2. As it is ineffective on the final results, the chromosomes can be adopted randomly. That is, regardless of chromosome, it tends to the optimum solution. The number of iterations is fixed (100).

The average of experiments on 200 packets is pictured in Figures 3(b) and 3(c). In the latter, the network sustains energy shortage at 0:39:15 due to heavy packets, while the ascending and continuous rate of packet transfer is evident until 5:10:24 in the former. The figures confirm the optimum placement, so that the network tolerates heavy packets optimally. In case of death of one node, the adjacent node is still almost functional for sending the data to the sink. Thus, the proposed algorithm is optimal and improves lifetime of the network [15].

## 4. Network Coverage in WSN

Coverage of WSNs has received great deal of attention in recent researches. The term is usually defined as a measure of performance of lifetime of the sensors in observing the physical space. The coverage is also a critical factor for connectivity of sensor network. By definition, connectivity is the capability of the sensor nodes to communicate with data sink. To deal with the issue of coverage, based on real-world WSN application, a set of hypothetical parameters (*A*, *B*, *C*) were assumed in a 2-D field. Consistent with other studies, the parameters are defined as close as possible to practical situation [12]. As a result, there are three types of sensors monitoring special objectives. For sake of more simplicity of the problem, the presumption is that spatial variables *A*, *B*, and *C* represent density of sensors per area that monitor objectives in the form of *ρ*_*A*_ ≪ *ρ*_*B*_ ≪ *ρ*_*C*_ correspondingly. In addition to general aspects of networks, the concept explains specific features of special-objective networks as well.

**Figure 4:**
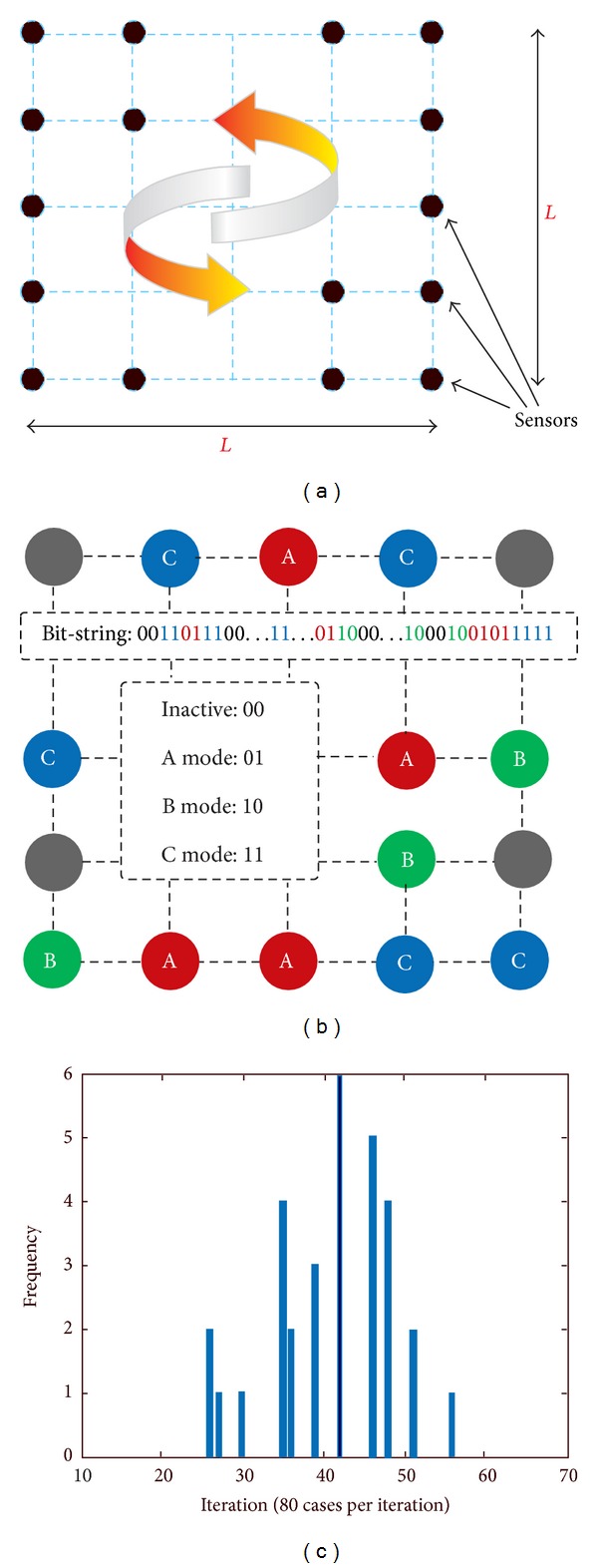
(a) Coverage scheme in wireless sensor network. (b) Network with represented encoding. (c) Network lifetime in specified scale.

A Euclidian square field at the length of 1 comprised of identical square area was assumed, so that all the subareas have sensor coverage located at the vicinal intersection lines. The configuration has been adopted in other works as a grid based wireless sensor network layout [12].  Figure 4(a) shows the general scheme of coverage in WSN.

The small sensors are featured with limited-power, limited range of transmission, and sensing mode option (three operating modes) based on capabilities and condition. With lower density of the parameter *A*, sensor is featured with the longest transmission range and *C* with the shortest range. To achieve optimum energy consumption, a clustering solution, with clusters consisting, one specific adjoining sensor of and the same operating mode known as cluster-in-charge, was devised. All the clusters may use multi-hop to communicate to the base station (BS) or sink. In normal operation, a cluster in charge carries out environment monitoring and data aggregation at specific periodic time and transmits the data to the BS. Here, the multiobjective algorithm capable to optimize the three main parameters (connectivity, consumption of energy, and coverage (ECEP)) of monitoring and measuring at required spots is introduced.

### 4.1. Proposed Fitness Function

To introduce some feasible optimum network topologies with as few as possible constraints (e.g., operational energy, number of unconnected nodes, and cluster-in charge overlap error), a novel algorithm was adopted.

Considering fitness function that takes the whole operational modes in general feasible states, the technique assesses the applied parameters.

#### 4.1.1. Coverage Problem Formulation to ECEP

To find the proper fitness functions as a part of genetic algorithm, the formula introduce by Quintão et al. [17] was used; the formula is an improved version of Nakamura's formula [18], where “*A*” is given monitoring area, “*S*” is set of sensor nodes, “*D*” is a set of demanded points, “Ad” is a set of areas needed to be monitored by sensors, “NC” is penalty cost of lack of coverage for the needed point, “AE” is turning energy on, and “PC” is penalty costs of the path stretching from every node to BS (obtained via Dijekstra's algorithm for a processing phase which is dedicated to each node to differentiate expensive nodes). The variables of the model are

 
*x*_*ij*_ = 1 when node “*i*” covers demand point *j* and 0 otherwise,
 
*y*_*i*_ = 1 when nodes “*i*” is active and 0 otherwise,
 
*h*_*j*_ = 1 when demand point “*j*” is not covered.
  (9) $$\begin{array}{c} \min\;\sum_{i\in S}\left(\text{A}{\text{E}}_{i}+\text{P}{\text{C}}_{i}\right)\times y_{i}+\sum_{j\in D}^{}\text{N}{\text{C}}_{j}\times h_{j}, \end{array}$$

subject to

(10) ∑ij(xij+hj)≥1, ∀j∈D  &  ∀ij∈Ad,xij≤yi, ∀i∈S  &  ∀ij∈Ad,0≤xij≤1, ∀ij∈Ad,  hj≥0, ∀j∈D,          y∈{0,1},  {x,h}∈ℜ.

The formula (9) enables us to have minimum required active nodes (more energy to the network) and minimum cases of lack of coverage as well. Every demanded point for monitoring by a sensor or keeping uncovered is represented by constraints (10); they dictate that only active nodes are able of sensing, respectively.

By taking into account penalty cost of overlapping cluster-in-charge errors and consumption of energy marked by OPCE and EC, respectively, an improvement was made in the fitness function (FF). We have

(11) FF=min⁡(Usage_Cost+Penalty_Cost),UsageCost=∑i∈s(AEi+PCi+EC)×yi,PenaltyCost=∑j∈M∑k∈D(NCjk+OPCEjk)×hk,

subject to

(12) $$\begin{array}{c} M\in \left\{A,B,C\right\}. \end{array}$$

As a dependent to sensor's mode in the network, EC is measured numerically. Clearly, high communication range is obtained by sensor working in mode “*A*” featured with highest rate energy consumption. By assuming 4 and 2 times power usage comparing with “*C*” for mode “*A*” and “*B*”, respectively, we have for EC,

(13) $$\begin{array}{c} \text{EC}=\frac{4n_{A}+2n_{B}+n_{C}}{\sum_{i\in S}n_{i}}. \end{array}$$

Taking into account OPCE in FF, wasted energy for overlapping error in cluster-in-charge is obtained.

#### 4.1.2. Ascertaining Connectivity to ECEP

Plenty of optimum solutions are obtained by genetic based algorithm, though connectivity of the nodes is not taken into account. This presents outflow of collected data toward the BS. Kruskal algorithm was utilized to examine connectivity of network in the 2nd part of ECEP. The process proposed is comprised of four steps.

The network is assumed as graph *G* where an edge exists between vertices “*x*” and “*y*” in graph *G* when the maximum communication range between two particular nodes “*x*” and “*y*” exceeds the distance between “*x*” and “*y*.” By introducing the Kruskal algorithm, a minimum spanning tree (MST) is achieved, so that a shorter path between each two vertices for routing aggregated data is achieved.

The connectivity with specific shortest paths is achieved when the number of MST tree edges is the same as the number of vertices −1; otherwise, inactive nodes are activated (this explains shorter transmission range than communication distance for some nodes). Kruskal technique is used on newly activated and disconnected nodes. This results in formation of new lightest tree.

The shortest path between each disconnected node to the BS is obtained and the internal sensor nodes of the paths are added to the set *E*.

Any newly activated node not listed in *E* is turned deactivated. This helps preservation of network energy while the quality is the same.

Finally, one or two network typology(s) were developed based on the range of transmission of nodes and position of sensor node. The networks that realized maximum network coverage are characterized with optimum coverage and energy usage.

#### 4.1.3. Encoding

The proposed approach was implemented in a square field (*L*∗*L*) with virtually equal subareas. Each node is positioned at intersection of the subareas and obtains four expressions: (1) active (00), (2) mode *A* active (01), (3) mode *B* active (10), and (4) mode *C* active (11).  Figure 4(b) shows the network with encoding.

The whole nodes arrangement in the network resembles a chromosome. That is, each node represents gen and a set of gens in specific order creates a certain chromosome. In this way, *L* nodes in a network host 2*L*2 bits as mode of each node is showed by two bits and there are *L*2 gens in the network.

### 4.2. Evaluation and Simulation Results

Genetic algorithm technique includes set of chromosomes known as population which improves by generation process. To put it another way, inspired by the nature, the algorithm receives input data which are randomly collected by the primary population. When generation process is completed, the final population/result represents the optimum solution for the main problem. In general, all improvements made by the generation process are comprised of crossover, scoring, selection, and mutation function. The term crossover refers to productive function at specific rate where two different chromosomes mate to produce new generation. Among different methods of crossover, single point is under focus here.

As the most critical part of genetic algorithm, scoring or assigning fitness, on the other hand, employs FF for scoring. Specific weight is assigned to each chromosome depending on the content. This is to say that each chromosome is a solution developed through iterations. There is a direct relation between fitness value of chromosome and chance of surviving in some generations. The FF is a totally problem-based design and achieving intelligent fitness function to differentiate qualified people has been the main concern of the literature. Superior chromosomes are adopted by selection process to create a new population with mutation technique that permits specific chromosomes to enter the new generation. The stochastic nature of GA dictates that different solutions with variant performance are obtained in different runs of the algorithm. The proposed algorithm was implemented by WSN simulator and almost 100% coverage over the monitored area was realized.

Level of energy consumption and number of active nodes along with live packets over time are listed in Table 3. Only 18 packets were first delivered to BS and later the number increased to 83 packets in second 01:18.

Table 4 represents that the network died last time in 5:13:781. Moreover, there is a gradual decrease in number of live packets as number of active sensor converges to 0.

Role of number of nodes on genetic algorithm iteration on lifetime of the network is pictured in Figure 4(c). It is implied by iterations 50 and 57 that more number of individuals does not necessary result in better solution. That is, meeting stop criterion is enough to reach the optimum solution.

The results of simulation confirmed merits of relatively large number of sensors with low energy consumption over activating fewer numbers of sensors with higher energy consumption.

## 5. Clustering in WSN

As mentioned earlier, increase of lifetime and expansion and load balance are the main requirements of WSNs applications. Proper clustering using optimized techniques of clustering is an option to realize these goals.

Generally, the cluster based methods suit monitoring applications featured with necessity of nonstop stream of data from sensors [11]; this calls for reducing the costs of timely data message delivery by routing protocols. The Low Energy Adaptive Clustering Hierarchy (LEACH) protocol, for instance, employs a hierarchical approach for clustering the network. There is an adopted cluster head for managing each cluster. The cluster head is in charge of several tasks; first it is comprised of collecting data supplied from the members of a cluster on periodical basis. It aggregates the data after gathering them, so that redundancy among correlated values is dealt with [19]. The next main task assigned to a cluster head is to directly transmit the aggregated data to the base station. The transmission is conducted via a single hop. Creating a TDMA-based schedule is aimed to assign a time slot to each cluster—to be used for transmission is the main task. The cluster members learn by the schedule when the head cluster disseminates it. To minimize probability of collision among the sensors inside and outside a cluster, a code-division multiple access-based scheme is employed by LEACH nodes for communication.

**Figure 5:**
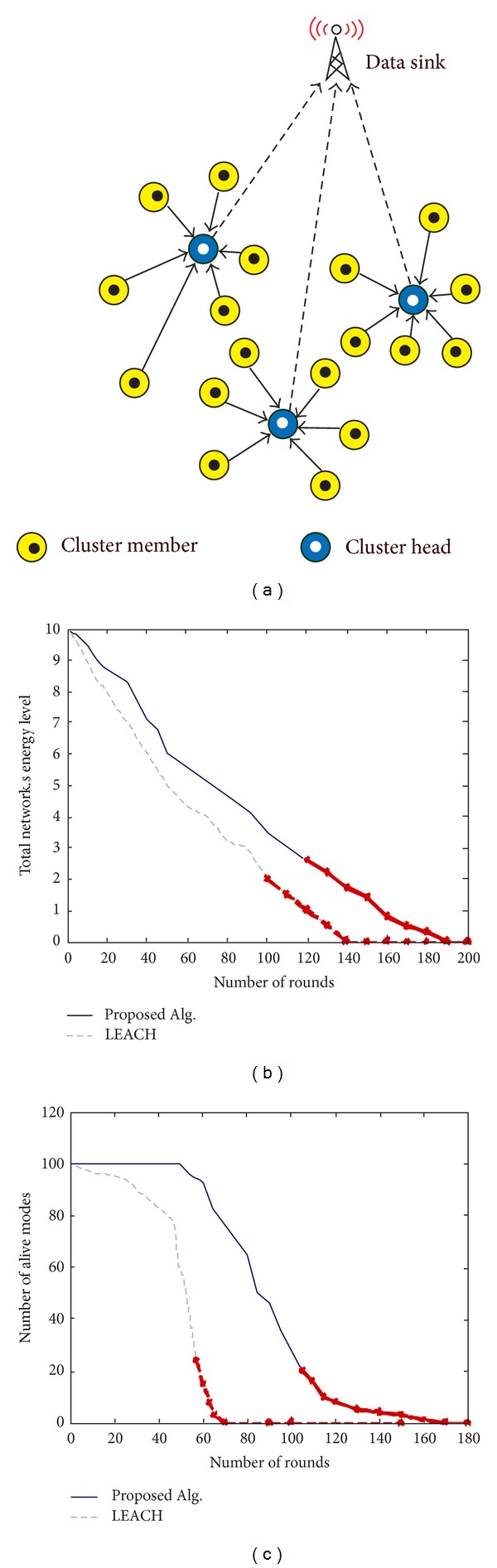
(a) A sample of cluster based WSN. (b) Energy consumption rate over the lifetime of a network. (c) Comparison of live nodes in two methods.

Figure 5(a) illustrates a sample of cluster based WSN. The aggregated data are transmitted from several clusters via CHs toward BS. The CHs are marked by gray circle. There are plenty of studies on protocols with less costs of transmission between CHs and BS.

### 5.1. The Proposed Fitness Function

For defining energy consumption and improve lifetime of the network, the parameters of the genetic algorithm were set according to software services. There is a negative relation between energy consumption and distance parameters. One way to lessen the distance between member nodes and pertinent CH is to use more clusters; each cluster may have one or more cluster head(s), which is not economic regarding the energy consumption. However, by using more clusters we avoid longer distances. Because of this, to achieve average amount of energy consumption by each node, a ratio of total energy usage to the total distances of nodes was defined. We propose a formula to achieve optimal WSN energy consumption and coverage (14).

Where, ((*e*_*i*_∗*T*) × *e*_*j*_∗*T*) is the total energy consumption and ((*D*_*a*_ × nodes)×(*D*_*b*_ × CHs)) is the total distance between nodes and each cluster is multiplied by total distance between cluster heads.  *F*(*i*) represents the maximum achievable value for the ratio. Taking into account the negative relation between number of cluster heads and number of nodes/CHs and the same relation between *e*_*k*_ and *e*_*j*_ on one hand and amount of *D*_*a*_ and *D*_*b*_ on the other hand, the maximum value of ratio action is a trade-off of energy consumption and number of blusters [17]:

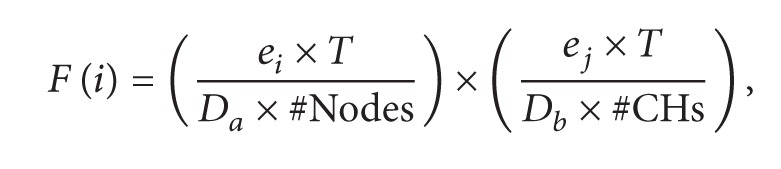
(14)

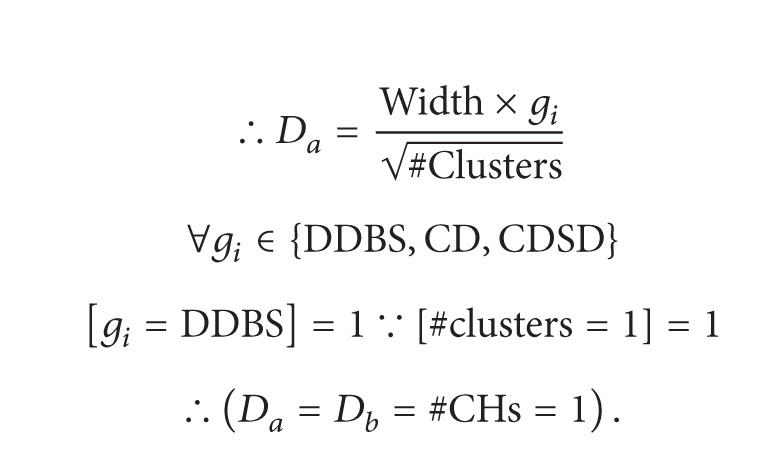
(15)
*F*(*i*) in the intelligent suitability function is capable of grading any chromosome both through cluster based method or direct method. To achieve the optimum solution, the optimum chromosome choice is made based on passing generation.

### 5.2. Evaluation and Simulation Result

A comparison is made between the GA-based approach proposed here and other cluster-based protocols (e.g., LEACH).

The parameters used in the simulation are listed in Table 5 and the clusters are featured with only one CH, while the generic algorithm process is used to obtain the number of CHs.

Table 6 represents the parameters of GA in the simulation. It is possible to adopt the chromosomes through random selection. The number of iterations is constant at 100.

A comparison between the proposed algorithm and LEACH regarding network energy and lifetime is pictured in Figures 5(b) and 5(c). The comparison is made over 200 periods of time. The former represents that unified consumption of energy by CHs results in short lifetime of nodes in LEACH. The latter shows that death of the first node in the proposed algorithm is delayed compared with LEACH as the node is removed due to energy status. Moreover, the network keeps working with minimum number of alive nodes. In general, the final individuals form a cluster with uniform energy consumption. This happens thanks to algorithm fitness function that takes the energy status of nodes and CHs/BS distances. In this way, the phenomenon adds to lifetime of the network significantly [20].

## 6. Data Aggregation in WSN

The purpose of data aggregation is to collect the highly critical data supplied by the sensors and to forward the data to the sink. Efficient energy consumption and reducing data latency as much as possible are two main concerns. The latter is vital for many applications including environment monitoring where fresh data are imperative. Achieving higher energy efficiency in data aggregation algorithm ensures longer network lifetime. Failing to share the load of data among the members of a network by the data aggregation tree eventuates in consumption of total energy by some of the nodes that are assigned with heavy load of data. Failure of nodes leads to failure of the network. Utilizing GA, this section investigates the data collecting spanning trees with higher energy efficiency. We try to achieve a proper route that balances the data load over the network. An algorithm that ensures a balance of residual energy among the nodes increases lifetime of the network.

The highest distance between every pair of nodes of the two clusters determines the distance between the clusters. MLDA is utilized on the basis of this cluster information. The (EESR) Energy-Efficient Sensor Routing was introduced by Hussain and Islam to be used on multi-hop network. To have higher efficiency of energy consumption, they used a spanning tree which is in fact a group of routing trees [21]. According to EESR, for calculating energy edge cost, the node with minimum energy is adopted. It uses the node with minimum energy and takes into account the lowest and highest cost links that receives data packets from the neighbor node and forward them to BS. Two spanning trees, featured with aggregation scheme and data gathering designed to guarantee higher lifetime of the network, were studied by Tan and körpeoğlu [22].

Yang and Fei proposed a new approach called Intermediate Target Based Geographic Routing (ITGR) to avoid such long detour paths. The novelty of the approach is that a single forwarding path can be used to determine a shaded area that may cover many destination nodes. They designed an efficient method for the source to find out whether a destination node belongs to a shaded area [23].

In general, there are two methods for power management among the nodes in data aggregation stage. One is the power aware version (PEDAPPA) that tries to achieve higher lifetime through creating balanced energy consumption by the nodes. The second method (PEDAP), the nonpower aware version, on the other hand, tries to attenuate energy consumption by the system on the basis of data gathering round [24]. The method ensures higher lifetime of the last node. Each method adopts different approaches to calculate the edge cost.

### 6.1. Problem Statement

The first assumption is that the network is initialized with every node having a fix range of radio communication and a specific primary energy before receiving the multi-data packet. All nodes are capable to monitor the environment, to send children packets to the neighbors, and to send single one to the corresponding parents. This process is performed as long as possible. According to the proposed algorithm in this part, every node, after initialization, may send a sample certain packet to the BS. In case a route is adopted for transmitting data packet, the BS utilizes a routing table with all the properties and current energy of the nodes listed to search for the monitor node with corresponding property and sends the resultant information to GA.

To find out an optimum route, the GA generates all possible routes. Afterward, BS prepares a schedule based on the route and sends it to all nodes. Then, the routing table is updated once more by GA while applying the reduction of energy for all the nodes. In effect, GA minimum spanning tree and aggregation tree are alike as the former is based on the environment-monitor node developed to examine the best edges toward the BS and to achieve balanced load of data packets to the nodes. In this case, the network is considered alive as far as the minimum required nodes are active to send data packet.

### 6.2. Fitness Function

Under fitness function, every chromosome is scored. This lets us to make comparison regarding number of deaths or survival over all the members. A formula later improved to be known as Nakamura formula was used in development of our proposed fitness function. Under the formula, “*A*” stands for the given monitoring area, “*S*” stands for the set of sensor nodes, “*D*” stands for the group of demanded points, “Ad” stands for the set of sensors in charge of monitoring the area under consideration, “AL” stands for the turning energy on in low energy level mode, “AH” stands for turning energy on in high energy level mode (sending packet load to the just “BS” stands, “EC” stands for the cost of a node in 3 states (*A*, *B*, *C*), and “*S*” stands for the set of edge collection Fitness function that is a procedure which scores any chromosome. This value helps us to compare the whole ones to each other for survival or death (15). The model can be formulated as

(16) G(i)=∑i∈S(AEi+ECi) ∀i∈D & ∀ii∈Ad,           AE∈{AL⁡,AH}.

The formula above (16) obtains all the feasible paths and the nodes' status. This information is required to set a node in low/high level energy mode. The proposed fitness function was improved by defining at least 3 states for all the node for obtaining the almost exact fitness as

(17) $$\begin{array}{c} \text{EC}=\frac{4n_{A}+2n_{B}+n_{C}}{\sum_{i\in S}n_{i}}\\ M\in \left\{A,B,C\right\}. \end{array}$$

The mode of the sensor network affects the EC, which is measured numerically. Knowing high range of communication for the sensor node in mode *A*, the highest energy consumption is expected under this mode. Modes *B* and *C*, on the other hand, with shorter communication range are next in order of energy consumption. It is assumed that energy consumption under mode *A* is four times more than that of *C*, and that of *B* in turn is 2 times more than that of *C*. EC is obtained through [25].

In the fitness function below, *N* stands for number of nodes; the function yields the electrical power based on setup energy. The energy needed for sending data packet from the children is marked by *E*_children_:

(18) $$\begin{array}{c} E{\left(i\right)}_{\text{Total}}=E_{\text{Monitor}}+G\left(i\right),\\ F\left(i\right)=\frac{E{\left(i\right)}_{\text{Total}}}{N}. \end{array}$$

The formula above gives the average energy mount through dividing by the number of nodes. The selection function is used to assess each individual, so that the better the fitness value, the more the chance for surviving to the next generation.

### 6.3. Evaluation and Simulation Results

A network simulator was used for implementation of the algorithm. The simulation is featured with two steps; first, Java editor is utilized in implementation of genetic algorithm based portion. To this end, Java Genetic Algorithm package (JPAC) was installed—there were other studies using the same method. Afterward, OMNET++ was employed to track the different routes between sensor node and BS in some simulated environment.  Table 7 lists the parameters for the experienced sensor network.

As Table 7 represents, each tree in the simulation is employed for 15 periods and the simulation is repeated 5 times for each scenario to obtain an average to report.

Parameters of the simulation of the environment are listed in Table 8. Taking into account priorities such as remaining energy and packet size, the simulation found all routes and GA adopted the optimum one out of them. Afterward, all nodes in the selected routes were scheduled by BS. Comparing with the other studies, the simulation showed that the proposed fitness function met the objectives.

**Figure 6:**
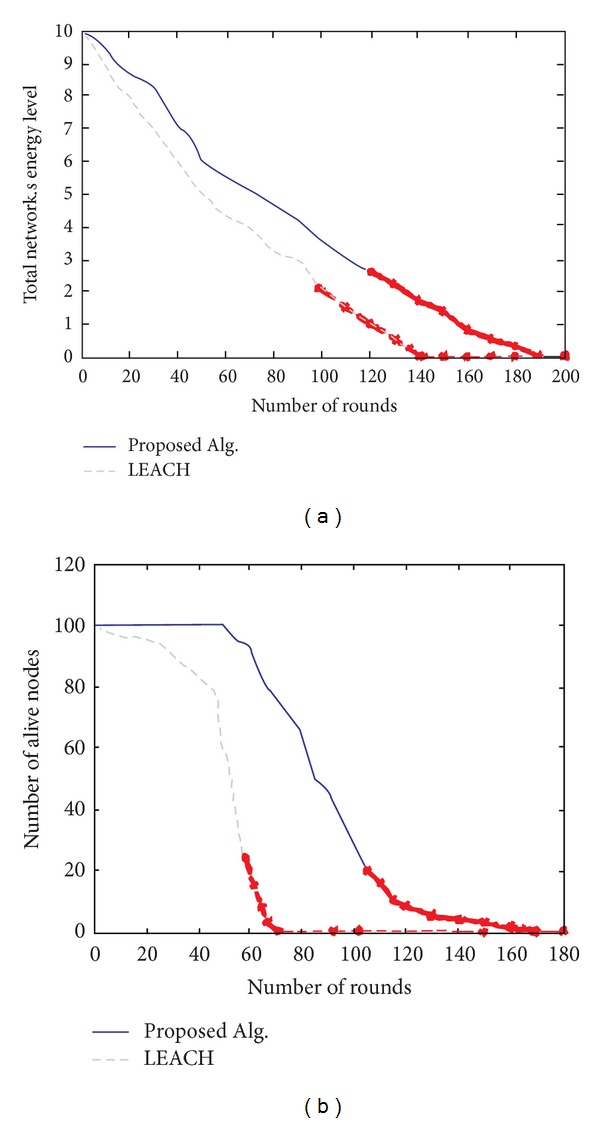
(a) Energy consumption rate in lifetime of virtual environments. (b) Comparison of coverage in two methods.

Comparisons between the proposed algorithm here and LEACH protocol on network energy and lifetime of 200 periods of time (year) are illustrated in Figures 6(a) and 6(b).  Figure 6(a) shows the unified packet load and energy consumption for obtaining the optimum route toward BS. On the other hand, Figure 6(b) pictures the time and the first node is removed. Clearly, in comparison with LEACH protocol, death time of the first node is considerably delayed. In addition, the network can keep working with minimum number of active node. In general, by using routing algorithm, fitness function takes three states of energy status (low, middle, and high) or communication range for the BS. The final individuals provide route with almost uniform energy consumption. This feature makes a great contribution to the network's lifetime [25].

## 7. Conclusion and Future Work

WSNs are comprised of a set of wireless sensors with variety of capabilities and limitations, which make them suitable for specific applications. There are several imaginable applications for WSNs in military, commercial, and medical fields. Taking into consideration the recent technological advances, utilization of these networks in daily life is increasing. Of the main limitations of WSNs is energy consumption and lifetime of the network, which are common concerns almost for any WSN application. In general, the operational stages of WSNs include node placement, network coverage, clustering, data aggregation, and routing. A technical survey was conducted on these operational stages. By finding the drawbacks and optimizing them, ideal parameters of the network were achieved. Finally, using genetic algorithm, a fitness function with optimum formula was obtained and the present protocols were optimized. The results of simulations in JPAC, MATLAB, and NS were compared with are of the present protocols and optimization of the two parameters confirmed. It is also noticeable that the diagrams obtained from the simulations showed an improvement in energy consumption parameters and lifetime of the network; this means more ideal WSNs. An application based protocol without specific limitation regarding its application—suitable for military, medical, and commercial applications—will be subject of our future studies.

## Figures and Tables

**Table 1:** Simulation parameters.

Network size	100 m
Node no.	200
Initial energy	2 J
*E*_*e*_	50 nJ/bit
ε_*l*_	0.0013 pJ/bit/m^2^
ε_*s*_	10 pJ/bit/m^2^
Network area	100 ∗ 100 m^2^
BS distance	200 m
Packet size	200 bits
*d*_co⁡_ = *d*_crossover_	85 m

**Table 2:** GA parameter values.

Number of candidate individuals	100
Length of chromosome	20
Crossover rate	.5
Mutation rate	.2
Iteration	100

**Table 3:** Observed values in the early times of network.

Time (nanosecond)	Power	Active sensors/35	Live packets
00:35.090	20718	27	18
00:49.330	15332	27	60
01:08.107	8031	20	53
01:18.182	6739	18	83
01:36.719	5333	14	30
01:48.626	4368	12	36
02:02.956	3354	10	38
02:09.666	3123	8	38
02:18.048	2897	8	47
02:46.709	2032	7	54
02:55.352	1860	6	52

**Table 4:** Observed values in the last times of network.

Time (nanosecond)	Power	Active sensors/35	Live packets
03:22.821	1096	5	87
03:45.804	616	4	89
03:58.382	421	3	75
04:15.196	307	2	33
04:39.882	122	1	25
05:13.781	0	0	0

**Table 5:** Simulation parameters.

Network size	100 m
Node no.	200
Initial energy	2 J
*E*_*e*_	50 nJ/bit
ε_*l*_	0.0013 pJ/bit/m^2^
ε_*s*_	10 pJ/bit/m^2^
Network area	100 ∗ 100 m^2^
BS distance	200 m
Packet size	200 bits
*d*_co⁡_ = *d*_crossover_	85 m

**Table 6:** GA parameter values.

Number of candidate individuals	100
Length of chromosome	20
Crossover rate	.5
Mutation rate	.2
Iteration	100

**Table 7:** Simulation parameters.

Network size	100 m
Node no.	200
Initial energy	0.8 J
BS location	Center of resource
Network area	100 ∗ 100 m^2^
Scenario simulated	5 times that average one is reported
Tree used time	15 periods

**Table 8:** GA parameter values.

Number of candidate individuals	500
Length of chromosome	20
Crossover rate	.7
Mutation rate	.7
Iteration	200
